# A new pathological scoring system by the Japanese classification to predict renal outcome in diabetic nephropathy

**DOI:** 10.1371/journal.pone.0190923

**Published:** 2018-02-06

**Authors:** Junichi Hoshino, Kengo Furuichi, Masayuki Yamanouchi, Koki Mise, Akinari Sekine, Masahiro Kawada, Keiichi Sumida, Rikako Hiramatsu, Eiko Hasegawa, Noriko Hayami, Tatsuya Suwabe, Naoki Sawa, Shigeko Hara, Takeshi Fujii, Kenichi Ohashi, Kiyoki Kitagawa, Tadashi Toyama, Miho Shimizu, Kenmei Takaichi, Yoshifumi Ubara, Takashi Wada

**Affiliations:** 1 Nephrology Center, Toranomon Hospital, Tokyo, Japan; 2 Okinaka Memorial Institute for Medical Research, Tokyo, Japan; 3 Department of Nephrology, Kanazawa University Hospital, Ishikawa, Japan; 4 Nephrology Center, Toranomon Hospital Kajigaya, Kanagawa, Japan; 5 Department of Nephrology, Rheumatology, Endocrinology, and Metabolism, Okayama University Graduate School of Medicine, Dentistry and Pharmaceutical Sciences, Okayama, Japan; 6 Department of Pathology, Toranomon Hospital, Tokyo, Japan; 7 Department of Pathology, Yokohama City University School of Medicine, Kanagawa, Japan; 8 Department of Nephrology, Kanazawa Medical Center, Ishikawa, Japan; 9 Department of Nephrology and Laboratory Medicine, Kanazawa University, Ishikawa, Japan; Tokushima University Graduate School, JAPAN

## Abstract

**Background and objectives:**

The impact of the newly proposed pathological classification by the Japan Renal Pathology Society (JRPS) on renal outcome is unclear. So we evaluated that impact and created a new pathological scoring to predict outcome using this classification.

**Design, setting, participants, & measurements:**

A multicenter cohort of 493 biopsy-proven Japanese patients with diabetic nephropathy (DN) were analyzed. The association between each pathological factor—Tervaert’ and JRPS classifications—and renal outcome (dialysis initiation or 50% eGFR decline) was estimated by adjusted Cox regression. The overall pathological risk score (J-score) was calculated, whereupon its predictive ability for 10-year risk of renal outcome was evaluated.

**Results:**

The J-scores of diffuse lesion classes 2 or 3, GBM doubling class 3, presence of mesangiolysis, polar vasculosis, and arteriolar hyalinosis were, respectively, 1, 2, 4, 1, and 2. The scores of IFTA classes 1, 2, and 3 were, respectively, 3, 4, and 4, and those of interstitial inflammation classes 1, 2, and 3 were 5, 5, and 4 (J-score range, 0–19). Renal survival curves, when dividing into four J-score grades (0–5, 6–10, 11–15, and 16–19), were significantly different from each other (p<0.01, log-rank test). After adjusting clinical factors, the J-score was a significant predictor of renal outcome. Ability to predict 10-year renal outcome was improved when the J-score was added to the basic model: c-statistics from 0.661 to 0.685; category-free net reclassification improvement, 0.154 (-0.040, 0.349, p = 0.12); and integrated discrimination improvement, 0.015 (0.003, 0.028, p = 0.02).

**Conclusions:**

Mesangiolysis, polar vasculosis, and doubling of GBM—features of the JRPS system—were significantly associated with renal outcome. Prediction of DN patients’ renal outcome was better with the J-score than without it.

## Introduction

Diabetic nephropathy (DN) is one of the main causes of end-stage renal disease (ESRD)—and probably among the most challenging kidney diseases—in many countries worldwide [1]. To control disease progression and establish new prognostic biomarkers, clarifying the association between a pathological change in DN and disease progression is essential.

Usually, DN is considered a main complication of diabetes when a patient with albuminuria has a history of diabetes longer than 5 years with no or mild hematuria. In daily clinical practice, renal biopsy is sometimes helpful for patients who have only short histories of diabetes, have no diabetic retinopathy, or do have massive hematuria, since it is known that earlier intensive treatments can prevent progression of DN [2, 3]. Moreover, typical findings of DN—e.g., mesangial expansion, arteriolar hyalinosis, and arteriosclerosis—are sometimes observed even in diabetic patients with normo-albuminuria [4, 5]. Reports also exist of various histopathological findings observed in patients with microalbuminuria [6, 7]. We therefore suggest that renal biopsy has some potential for assessing various kinds of kidney tissue damage and considering DN prognosis.

Recently, Tervaert et al. developed a new pathological classification for DN [8], and we created a new DN pathological scoring system for this classification to predict renal outcome [9] and clarify the importance of both interstitial damage and glomerular change [10–12], the latter’s classification based on the findings of mesangial expansion and nodular lesion. It has also been reported that there are many other typical glomerular changes in DN patients, such as masangiolysis, doubling of glomerular basement membrane, polar vasculosis, and glomerulomegaly [13–16]. Accordingly, the Japan Renal Pathology Society (JRPS) recently proposed a new DN classification and reported the association of those pathological changes and their outcomes [17]. However, it remains unknown how much those pathological changes impact overall renal outcome, and without that knowledge, it seems impossible to discuss the indications for renal biopsy. Furthermore, we must clarify the impact of each pathological change on renal outcome in order to understand the mechanism of disease progression. Therefore, in this study, we aimed to create a new pathological scoring system using a new Japanese classification for DN patients allowing prediction of the patient’s renal outcome.

## Materials and methods

### Study population

All patients with diabetes mellitus type 2 at Toranomon Hospital, Toranomon Hospital Kajigaya, Kanazawa University, and Kanazawa Medical Center who underwent renal biopsy and were confirmed with pure DN diabetes from February 1985 to March 2013 were enrolled and followed until ESRD, death, or end of follow-up. We reviewed original biopsy reports, and all biopsy specimens were confirmed to show pure DN, defined as DN without coexisting renal disease and without kidney transplantation. Patients with eGFR <10 mL/min/1.73m2 at renal biopsy or those whose obtained glomeruli were <5 were excluded. The median and interquartile range (IQR) of the follow-up year was 4.6 (1.8, 9.9) years.

We divided the cohort into a training set for developing the scoring system and a test set for validation. Those from Kanazawa Medical Center were used as the test set because they lacked information about urinary red blood cells (RBC) and presence of diabetic retinopathy. The protocol of database creation was approved with a waiver of the requirement to obtain informed consent by the ethics committees of Toranomon Hospital (Approval No.746) and Kanazawa University (Approval No. 1204–2). The database was anonymized and de-identified before analyses. The procedures fully adhered to the Declaration of Helsinki, and the Strengthening the Reporting of Observational Studies in Epidemiology.

### Clinical and laboratory investigations

Medical records provided patients’ baseline clinical data including age, sex, body mass index (BMI), eGFR, blood pressure (BP), hemoglobin, total cholesterol, serum albumin, duration of diabetes, hemoglobin A1c (HbA1c), albuminuria, urinary RBC, and presence of diabetic retinopathy. All laboratory values were measured using automated standardized methods at each hospital within 24 hours after drawing blood and urine samples. The eGFR was calculated using a formula for Japanese patients devised by Matsuo et al. [18]. Baseline age, eGFR, BMI, and HbA1c were treated as continuous variables. Albuminuria at baseline was measured in a 24-hour urine sample or a spot urine sample, and was treated as a categorical variable by classifying the samples as normo-albuminuria (<30 mg/day or mg/g creatinine(gCre)), micro-albuminuria (30-<300 mg/day or mg/gCre), macro-albuminuria (>300 mg/day or mg/gCre, or proteinuria > 0.5 g/day or g/gCre), or nephrotic range (proteinuria ≧3.5 g/day or g/gCre). Urinary RBC were considered positive if urinary RBC sediment ≧10/high power field in at least two of three consecutive urine specimens. HbA1c data are presented hereafter as National Glycohemoglobin Standardization Program values [19]. Mean BP was calculated by doubling the diastolic BP, adding the sum to the systolic BP, then dividing by three.

### End point

The primary end point was defined as a decline in eGFR of ≧50% from baseline or commencement of dialysis because of ESRD. None of patients received kidney transplantation during follow-up.

### Histopathological diagnosis

All renal biopsy specimens were obtained by percutaneous needle biopsies based on decisions by our department and/or primary nephrologists. Generally, standard indications for renal biopsy were unexplained proteinuria (≧0.5 g/day) including nephrotic syndrome with short diabetes history, or presence of urinary RBC. All specimens were evaluated at the pathological laboratories of Toranomon Hospital and/or Kanazawa University using light microscopy, immunofluorescence, and electron microscopy—evaluation made by at least two renal pathologists and/or nephrologists in accordance with the two criteria of diabetic nephropathy from the Renal Pathology Society (RPS) and from JRPS (S1 Table) [8, 17].

### Statistical analyses

Data were summarized using proportions, means with standard deviation (SD) and medians with IQR as appropriate. Hazard ratios (HR) of renal outcome for each stage of JRS pathological variables were estimated by Cox proportional hazards regression model after adjusting for clinical factors: age, sex, eGFR, mean BP, BMI, HbA1c, albuminuria, urinary RBC, and presence of diabetic retinopathy. Because the correlation coefficient was high between pathological variables, HRs of stages in each pathological variable were separately analyzed to avoid multicollinearity.

To weight the relative importance of each pathological variable for renal outcome, bootstrap inclusion fractions (BIF) were calculated by bootstrap aggregating with 500 resampling [9, 20–22]. To generate a simple integer-based point score for each pathological variable stage, scores were given by multiplying the beta coefficient by 5, multiplying by the estimated BIF of the pathological variable, and rounding to the nearest integer. The net pathological score (Japanese diabetic pathological score, i.e., J-score) for each patient was calculated by summing the scores of all components. The calculated J-score was divided into four groups. The cut-off value was evaluated by comparing the incidence rates for renal outcome of each score. To evaluate how well the scoring system fit, we compared renal survival curves in each group using Kaplan-Meier and the log-rank test. The D-score derived from the RPS classification was also calculated for further comparison [9].

Predictability of renal outcome within 10 years when D-score or J-score was added to the baseline clinical model was assessed by C-statistics, category-free net reclassification improvement (NRI), and integrated discrimination improvement (IDI). The model 1 included eGFR and albuminuria, and the model 2 included age, sex, eGFR, mean BP, BMI, HbA1c, and albuminuria. For all analyses, two-tailed p-values <0.05 were considered significant. All analyses used Stata® SE version 14.1 (StataCorp, College Station, TX.).

## Results

### Baseline characteristics

Table 1 shows the study population’s baseline characteristics stratified by training and test sets. Renal outcome (initiation of dialysis or 50% reduction of eGFR) was observed in 56% of our 493 patients (68% men; mean age, 57.2±12.3 years; mean eGFR 52.7±28.7 ml/min/1.73m^2^; normo-albuminuria 9%, micro-albuminuria 17%, macro-albuminuria 46%, and nephrotic, 26%) with mean follow-up of 5.1±5.9 years. The 326 patients treated at Toranomon Hospital, Toranomon Hospital Kajigaya, and Kanazawa University were a training set for score development while the 167 patients at Kanazawa Medical Center were a validation set. Table 1 shows that patients’ characteristics in the test set were slightly different from those of the training set: higher age, lower BMI, better CKD stage, and higher proportions of death or renal outcome.

**Table 1:** Baseline characteristics (n = 493).

Clinical findings		Total	Training set	Test set	p
**n**		**493**	**326**	**167**	
Male		68%	71%	63%	0.11
Age (years)		57.2±12.3	55.7 ± 12.8	60.2±10.7	<0.001
Body mass index (kg/m^2^)		23.7±3.9	24.1 ±4.0	22.8±3.4	0.002
Systolic BP (mmHg)		144±21	145±20	142±22	0.14
Diastolic BP (mmHg)		79±13	81±13	76±12	<0.001
eGFR (ml/min/1.73m^2^)		52.7±28.7	52.7±28.7	55.3±33.8	0.14
Heatmap	G1	10%	7%	15%	0.02
	G2	27%	29%	22%	
	G3a	20%	19%	21%	
	G3b	19%	21%	17%	
	G4	18%	19%	15%	
	G5	7%	5%	10%	
Albuminuria (mg/gCre)					
normo (<29)		9%	9%	7%	0.003
micro (30–299)		17%	14%	23%	
macro (300+)		46%	46%	52%	
nephrotic		26%	30%	17%	
Hemoglobin (g/dL)		12.0±2.3	12.2±2.3	11.6±2.5	0.02
Hemoglobin A1c (%)		7.7±2.1	7.8±2.0	7.6±2.1	0.25
Total cholesterol (mg/dL)		218±73	219±69	215±80	0.55
Urinary RBC (%)			12%	-	
Albumin (g/dL)			3.1±0.7	-	
Retinopathy (%)			70%	-	
DM duration (years)			13.1±8.6	-	
Death		64 (14%)	26 (8%)	38 (27%)	<0.001
Follow-up (year)		6.7±6.4	6.3±6.3	7.7±6.4	0.03
Renal outcome		261 (56%)	174 (53%)	87 (64%)	0.04
Follow-up (year)		5.1±5.9	4.9±5.8	5.6±6.3	0.22

Abbreviation: eGFR, estimated glomerular filtration ratio; Renal outcome, initiation of dialysis or 50% eGFR decline

All biopsy specimens were evaluated by both the RPS and JRPS systems. S2 Table shows that the proportion of patients having particular diabetic glomerular lesions in the whole set was: nodular lesion, 36%; GBM doubling, 40%; mesangiolysis, 39%; polar vasculosis, 69%; glomerulomegaly, 30%; and exudative lesion, 62%. S2 Table also shows distribution of interstitial fibrosis and tubular atrophy (IFTA), interstitial inflammation, arteriolar hyalinosis, and arteriosclerosis. Distribution of classes 0 and 1 in both interstitial inflammation and arteriolar hyalinosis was very similar among JRPS and RPS patients. Therefore, it seems that the JRPS divides RPS class 2 into classes 2 and 3. We also found that most of the glomerular lesions (more severe diffuse lesions, nodular lesions, and masangiolysis), more severe IFTA, and arteriolar hyalinosis became prominent (≧25%) in CKD heatmap orange or red. Also, polar vasculosis (21%), exudative lesions (17%), interstitial inflammation (RPS grade 2, 19%), and arteriosclerosis (grade 2, 14%) were observed even in CKD heatmap green. Comparing the datasets, the proportion of patients having GBM doubling, mesangiolysis, glomerulomegaly, arteriolar hyalinosis, and arteriosclerosis was higher in the training set than the test set, while the proportion of those with polar vasculosis and exudative lesion was lower (S2 Table).

Next, we checked the correlation between clinical and pathological factors. As shown in S3 Table, many of the factors were highly correlated, with the highest correlation between nodular lesions and mesangiolysis (r = 0.6018). In particular, diffuse lesions were correlated with most of the other pathological factors, and eGFR was highly correlated with IFTA (r = -0.5684), interstitial inflammation (r = -0.4485), and urinary albumin (r = -0.4165).

### Pathological factors associated with renal outcome

We then evaluated HRs for renal outcome of DN patients in each pathological class after adjusting for age, sex, BMI, eGFR, mean BP, HbA1c, grade of albuminuria, urinary RBC, and presence of DM retinopathy. Since there were strong correlations (r>0.5) between each pathological factor, we included pathological factors as separate items in the Cox proportional hazard model for analyzing factors associated with renal outcome. Table 2 shows that the presence of diffuse lesions class 2 (vs class 0), GBM doubling class 3 (vs class 0), nodular lesion, mesangiolysis, IFTA classes 1–3 (vs class 0), interstitial inflammation classes 1–3 (vs class 0), and arteriolar hyalinosis classes 2–3 (vs class 0) were significantly associated with renal outcome.

**Table 2:** HR, BIF, and score in each JRPS pathological class.

	Hazard ratio	*95% CI—Lower*	*95% CI* *- Higher*	p-value-	β coef- ficient	BIF	score
**Diffuse lesion 0**	1.00						
**1**	2.83	*0*.*91*	*8*.*83*	0.07	1.04	0.082	0
**2**	5.65	*1*.*78*	*17*.*96*	0.003	1.73	0.082	1
**3**	3.12	*0*.*97*	*10*.*02*	0.06	1.14	0.082	1
**GBM doubling 0**	1.00						
**1**	1.38	*0*.*91*	*2*.*11*	0.12	0.32	0.276	0
**2**	0.99	*0*.*56*	*1*.*73*	0.96	-0.01	0.276	0
**3**	2.88	*1*.*39*	*5*.*95*	0.004	1.06	0.276	2
**Exudative lesion**	1.37	*0*.*94*	*1*.*99*	0.10	0.31	0.074	0
**Nodular lesion**	1.62	*1*.*12*	*2*.*35*	0.01	0.48	0.128	0
**Mesangiolysis**	2.11	*1*.*41*	*3*.*16*	<0.001	0.75	0.984	4
**Polar vasculosis**	1.47	*0*.*98*	*2*.*19*	0.06	0.39	0.262	1
**Glomerulomegaly**	1.19	*0*.*84*	*1*.*69*	0.33	0.17	0.11	0
**IFTA 0**	1.00						
**1**	4.20	*1*.*53*	*11*.*59*	0.006	1.44	0.474	3
**2**	5.92	*2*.*03*	*17*.*29*	0.001	1.78	0.474	4
**3**	6.48	*2*.*15*	*19*.*51*	0.001	1.87	0.474	4
**Interstitial inflammation 0**	1.00						
**1**	4.69	*2*.*06*	*10*.*68*	<0.001	1.55	0.626	5
**2**	4.93	*1*.*97*	*12*.*34*	0.001	1.60	0.626	5
**3**	3.26	*1*.*23*	*8*.*64*	0.02	1.18	0.626	4
**Arteriolar hyalinosis 0**	1.00						
**1**	2.98	*0*.*95*	*9*.*29*	0.06	1.09	0.332	2
**2**	3.79	*1*.*23*	*11*.*68*	0.02	1.33	0.332	2
**3**	3.80	*1*.*24*	*11*.*63*	0.02	1.34	0.332	2
**Arteriosclerosis 0**	1.00						
**1**	0.74	*0*.*42*	*1*.*32*	0.331	-0.30	0.168	0
**2**	1.01	*0*.*55*	*1*.*85*	0.996	0.01	0.168	0

Abbreviations: CI, confidence interval; BIF, bootstrap inclusion fraction; IFTA, interstitial fibrosis and tubular atrophy

To calculate the pathological score, we gave a weight to each pathological factor and summed the products of the beta and that weight. We initiated the bootstrap aggregating method and calculated the BIF by assessing the weight of each pathological factor—as done previously. Table 2 shows the BIF of each lesion after bootstrapping 500 times. The component of J-score was calculated by multiplying the beta coefficient by 5, multiplying by the estimated BIF of each pathological variable, and rounding to the nearest integer. Scores of diffuse lesion classes 2 and 3 were 1, those of GBM doubling class 3, mesangiolysis, and polar vasculosis, arteriolar hyalinosis classes 1–3 were, respectively, 2, 4, 1, and 2. The scores of IFTA classes 1, 2, and 3 were 3, 4, and 4, and those of interstitial inflammation classes 1, 2, and 3 were 5, 5, and 4. So the possible range of J-score was 0–19.

**Fig 1:**
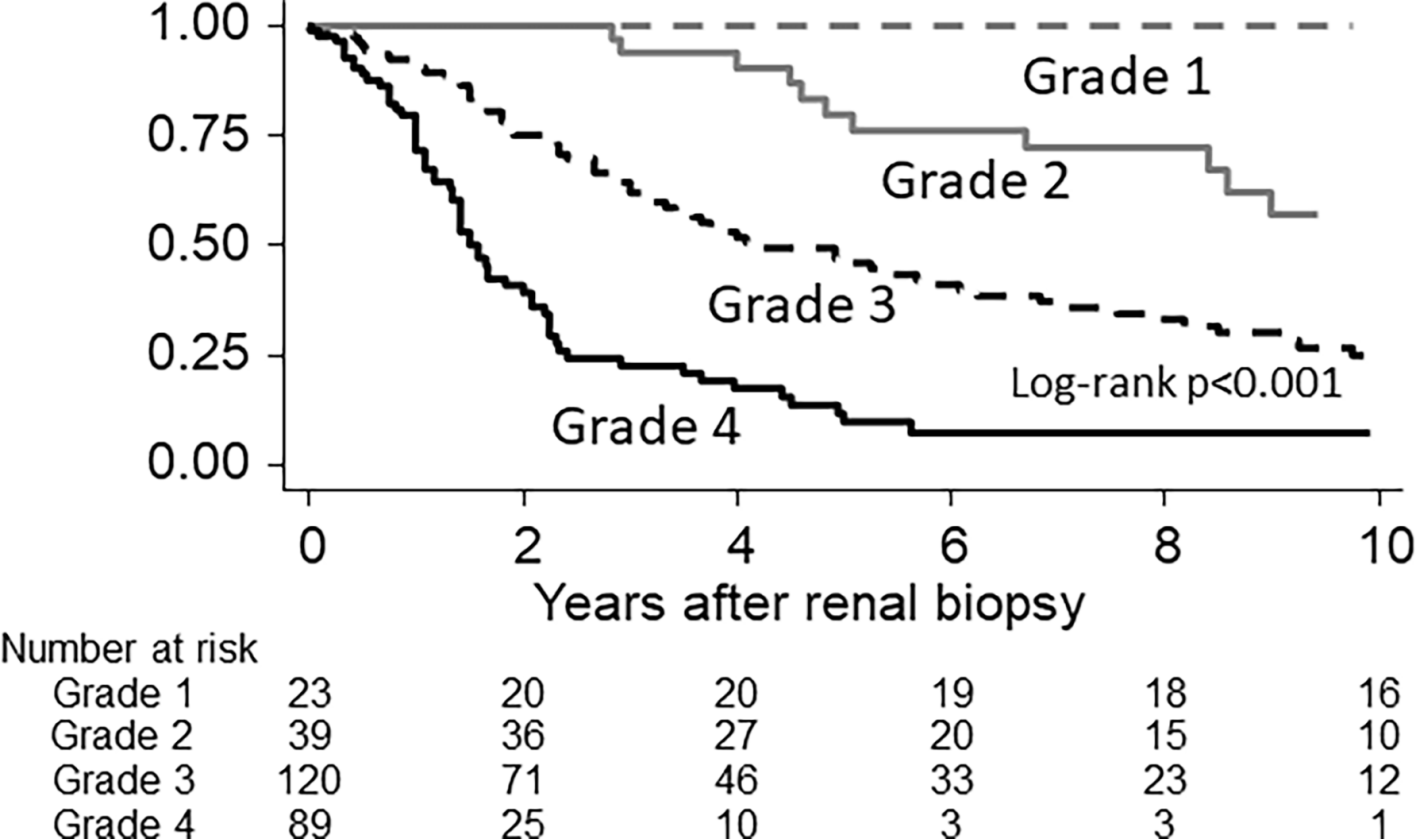
Renal survival after renal biopsy dividing by pathological score categories. Abbreviations: Grade 1, J-score 0–5; grade 2, J-score 6–10; grade 3, J-score 11–15; and grade 4, J-score 16–19.

Next, according to the 10-year risk of renal outcome, we divided patients into four groups (grades 1 to 4), those with J-score 0–5 (incidence ≦0.01 person/person-year), 6–10 (≦0.10), 11–15 (≦0.30), and 16–19 (>0.30). Renal survival curves after renal biopsy, when dividing into J-score grades, were significantly different from each other (p<0.01, log-rank test) (Fig 1). The IQR renal survivals in each grade were >18 years in grade 1, 12.5 (4.8–22.3) years in grade 2, 4.0 (1.9–9.9) years in grade 3, and 1.7 (1.0–2.4) years in grade 4. After adjusting for age, sex, eGFR, proteinuria, mean BP, BMI, HbA1c, DM retinopathy, albuminuria, and urinary RBC—and with ≦5 as reference—the HRs for renal outcome of patients with J-score 6–10, 11–15, and 16–19 were, respectively, 10.98 (2.32–51.90), 17.82 (3.88–81.91), and 36.47 (7.61–174.79) in the training set (Table 3). Therefore, in addition to clinical factors, the J-score seemed a significant predictor of renal outcome after renal biopsy.

**Table 3:** Factors affecting renal outcomes in patients with diabetic nephropathy (training set).

		Hazard ratio	95% CI		p-value
Age	per year	0.98	0.96	0.99	0.01
Gender	(male)	0.84	0.56	1.24	0.38
eGFR (per 10ml/min/1.73m^2^)		0.83	0.75	0.92	0.00
Mean Blood pressure	(mmHg)	1.02	1.00	1.03	0.03
Body Mass Index	(kg/m^2^)	0.98	0.94	1.03	0.49
Hemoglobin A1c	(%)	0.92	0.83	1.02	0.13
Diabetic retinopathy		0.80	0.52	1.23	0.32
Albuminuria	normo				
	micro	1.96	0.48	8.06	0.35
	macro	5.43	1.57	18.80	0.01
	nephrotic	11.02	2.94	41.35	<0.001
Urinary RBC		1.79	1.09	2.95	0.02
J-score	≦5				
	6–10	10.98	2.32	51.90	0.002
	11–15	17.82	3.88	81.91	<0.001
	16–19	36.47	7.61	174.79	<0.001

Abbreviation: CI, confidence interval; J-score: Japanese diabetic pathological score, eGFR: estimated glomerular filtration rate; RBC, red blood cell; Renal outcomes, 50% reduction of eGFR or dialysis initiation.

### Utility of the new pathological score by levels of albuminuria and renal function

**Fig 2:**
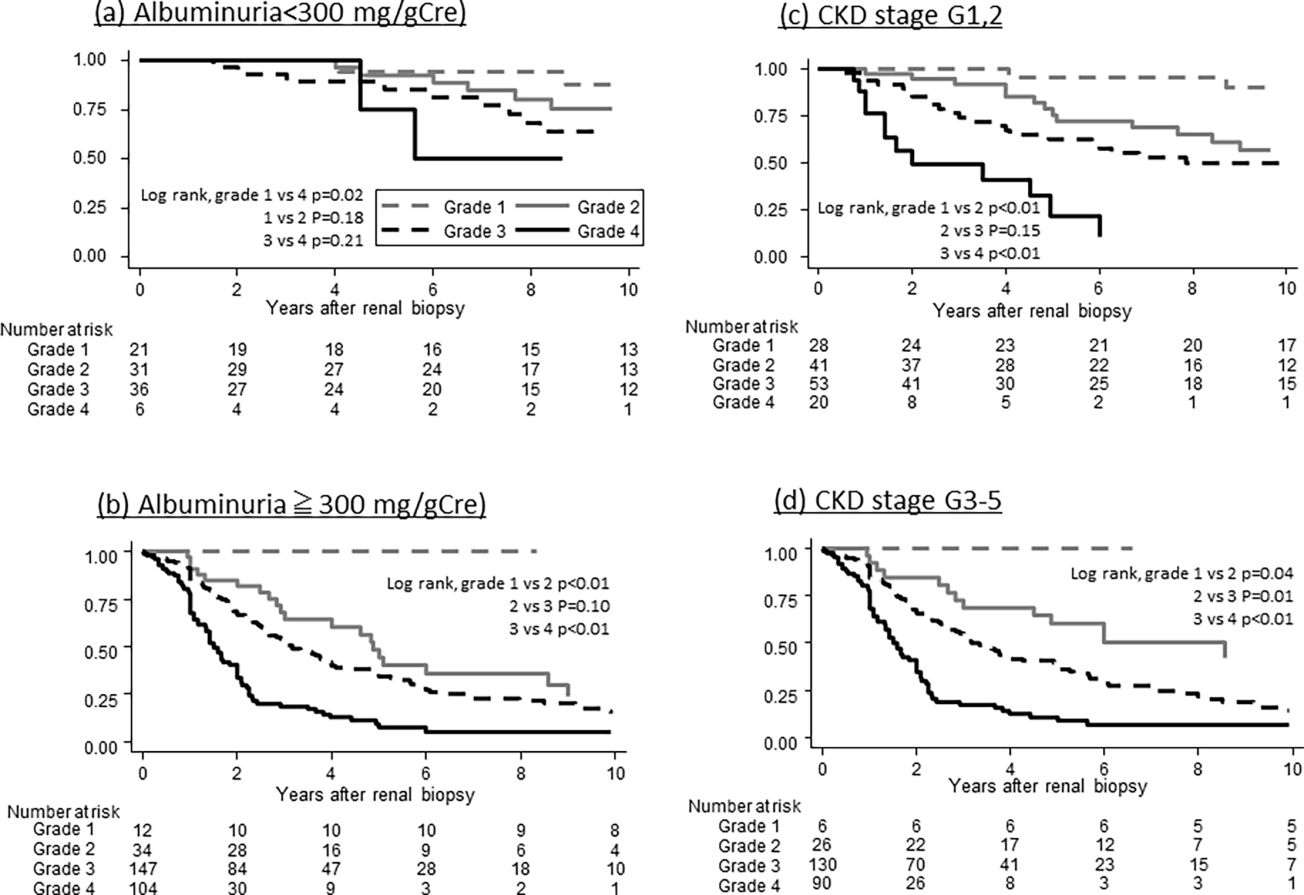
Renal survival after renal biopsy divided by pathological score categories and by level of urinary albumin and renal function. Abbreviations: Grade 1, J-score 0–5; grade 2, J-score; grade 3, J-score 11–15; and grade 4, J-score 16–19; CKD, chronic kidney disease.

Then we evaluated the heterogeneity of the new pathological score by the levels of albuminuria and renal function. The median and interquartile range of J-score in patients with normo-albuminuria, micro-albuminuria, and macro-albuminuria were significantly different among the groups (respectively, 8.5 [2–10], 11 [8–12], and 13 [11–16], p<0.001) (S4 Table), suggesting the heterogeneity of J-score even in the early stage of DN. Also, the score in patients with CKD G1/2, 3, and 4/5 were significantly different among the groups (respectively, 10 [7–13], 13 [11–16], and 14 [12–17], p<0.001) (S4 Table). Renal survival in patients with higher J-scores was significantly worse regardless of levels of albuminuria and renal function (Fig 2).

### Predictability of 10-year renal outcome when J-score is added

To validate the scoring system, we assessed its ability to predict 10-year renal outcomes in the test set when the J-score was added to the basic model and clinical model. The basic model included eGFR and albuminuria; the clinical model included age, sex, eGFR, mean BP, BMI, HbA1c, and albuminuria. Table 4 and S1 Fig. show that the basic model (model 1)’s c-statistics improved from 0.661 to 0.685 (0.634, 0.736) while NRI and IDI were, respectively, 0.154 (-0.040, 0.349) (p = 0.12) and 0.015 (0.003, 0.028) (p = 0.02). The clinical model (model 2)’s c-statistics slightly improved from 0.722 to 0.724 (0.672, 0.775) while its NRI and IDI were 0.194 (-0.010, 0.398) (p = 0.06) and 0.013 (0.001, 0.024) (p = 0.03). These results were consistent when we used the training set instead of the test set. In addition, as a sub-analysis, we checked the predictability of the J-score in patients with mild albuminuria (<300 mg/gCre), or with preserved renal function (eGFR≧60 mL/min/1.73m^2^). The basic model’s c-statistics improved from 0.616 to 0.679 (0.556, 0.802), and from 0.682 to 0.757 (0.679, 0.834) in patients, respectively, with mild proteinuria and with preserved renal function (S5 Table)—a finding similar to that of our original analyses. This therefore suggests that the predictability of 10-year renal outcome would be improved by adding the J-score to the clinical model regardless of levels of albuminuria or renal function.

**Table 4:** Ability to predict 10-year renal outcome with and without J-score.

**Model 1**	**C-statistics**	**NRI**	**IDI**
**Clinical**	0.661 (0.614, 0.709)		
**Clinical + J-score**	0.685 (0.634, 0.736)	0.154 (-0.040, 0.349), p = 0.12	0.015 (0.003, 0.028), p = 0.02
**Clinical + D-score**	0.751 (0.672, 0.830)	0.691 (0.355, 1.027), p<0.001	0.031 (-0.008, 0.062), p = 0.06
**Model 2**	**C-statistics**	**NRI**	**IDI**
**Clinical**	0.722 (0.674, 0.769)		
**Clinical + J-score**	0.724 (0.672, 0.775)	0.194 (-0.010, 0.398), p = 0.06	0.013 (0.001, 0.024), p = 0.03
**Clinical + D-score**	0.673 (0.571, 0.775)	0.539 (0.185, 0.893), p = 0.003	0.043 (-0.010, 0.398), p = 0.06

Clinical model 1: estimated glomerular filtration rate (eGFR) and albuminuria category

Clinical model 2: age, sex, eGFR, blood pressure, body mass index, hemoglobin A1c, and albuminuria category

NRI, net reclassification improvement; IDI, integrated discrimination improvement

Next, we compared the predictability of the J-score with that of the D-score, which was based on the RPS criteria [9]. Table 4 shows the c-statistics, NRI and IDI of models 1 and 2 when D-score was added. Most predictive markers improved when D-score was added to the clinical factors. Performance seems similar between J-score and D-score models.

## Discussion

We assessed prognostic values of the pathological factors in the new pathological classification recently proposed by the JRPS. Compared with the RPS classification, the JRPS classification evaluates the glomerular changes of DN in greater detail, including doubling of GBM, mesangiolysis, and polar vasculosis. Our study revealed the importance of evaluating the doubling of GBM, mesangiolysis, and polar vasculosis in the glomeruli of DN patients since it turned out that independent factors were associated with renal outcomes. Surprisingly, the impact of mesangiolysis on renal outcome in the J-score was equivalent to the impact of severe IFTA and interstitial inflammation, suggesting some potential superiority of JRPS over the RPS classification for assessing renal outcome in DN patients. However, the impact of diffuse lesion of the glomeruli themselves on renal outcome was relatively small, though it is one of the famous findings of DN.

We also created a new pathological scoring system based on the JRPS pathological classification. Fioretto et al. reported that pathological improvement of DN was not parallel among glomerular lesions and tubular-interstitial lesions after normalization of glycemic control achieved by pancreas transplantation [23]. Many previous papers have suggested the importance of evaluating tubule-interstitial lesions—not only glomerular lesions—in DN for renal prediction, which is consistent with previous findings [10–12, 24–26]. Our results highlighted the fact that the evaluation of tubule-interstitial changes may equal or exceed in importance evaluation of glomerular lesions in predicting renal prognosis. Therefore, an evaluation system that includes both glomerular and tubulointerstitial lesions is needed for considering renal prognosis. Since all pathological findings are highly correlated, it is essential to create some net pathological damage index caused by diabetes mellitus, which may make it easier to compare the prognostic value of pathological evaluation with that of clinical information.

The clinical importance of renal biopsy for DN is still debated. The KDOQI guideline 2007 reported that, generally, careful screening of diabetic patients can identify DN without renal biopsy, but that kidney biopsy may be required if normoalbuminuric patients have decreased GFR [27]. Recently, Tangri et al. developed a nice equation with only four clinical factors—age, sex, eGFR, and albuminuria—for predicting risk of kidney failure in a Canadian population with CKD grades 3–5. Its utility was validated in 31 multinational cohorts [28, 29]. Yet, other reports stressed the importance of histological evaluation even in the early DN stage because of wide pathological heterogenicity [4, 5, 30]. Our previous report suggested that the presence of nodular lesions, exudative lesions, and mesangiolysis in cases in the CKD heatmap categories green and yellow were associated with a great impact on composite kidney events (dialysis, doubling of serum creatinine, or reduction of eGFR by half) before and after adjustment for clinical risk factors[17]. Several other papers reported the importance of nodular lesions, exudative lesions, and mesangiolysis in predicting renal outcome[5, 10, 25]. In addition, to our knowledge, there is no predicting risk score of kidney failure in patients with CKD grades 1–2. So our scoring system may be helpful in predicting renal outcome in the early DN stage since we found a small but significant improvement in predicting renal outcome when we added pathological information. Since progression of DN is sometimes reversible by intensive treatments—especially in the earlier DN stage [3, 31, 32]—there is no doubting the importance of early DN detection. Although recent reports suggest the utility of biomarkers such as L-FABP, TNFR2, and Kim-1 [33–35] for early renal failure detection, pathological evaluation based on renal risk prediction is necessary to understand the association between pathophysiological change and biomarkers, because, to our knowledge, there are no definite clinical signs or positive biomarkers that accurately predict presence of nodular lesions, exudative lesions, or mesangiolysis. This scoring system may help evaluate that correlation, and may contribute, in some measure, to revealing the roles of biomarkers in renal tissue.

There were several limitations in this study. First, as expected with biopsy studies, selection bias may exist. But the consistency of findings among the two independent cohorts comprised of multicenter patients may minimize that bias. Moreover, this is the largest cohort ever evaluated by both RPS and JRPS classifications. Second, treatment effects during observation were not taken into account. However, because our four hospitals are Japan’s leading DN care institutions—staffed by experts—and given the high proportion of RAS inhibitor use regardless of cohort, it is reasonable to think that each patient received the best practice. In fact, when we performed sensitivity analysis only of patients who had treatment data during observation—such as average BP and HbA1c data—the results were consistent regardless of study population.

In conclusion, we evaluated the impact of the pathological changes of DN recently proposed by the JRPS, and discovered the importance of finding mesangiolysis, doubling of GBM, and polar vasculosis on predicting renal outcome. In addition, we created a new pathological DN scoring system to predict patients’ renal outcome. Based on this J-score system, we could, indeed, predict renal outcome; and we found that if the J-score is ≦5, the predicted renal outcome is excellent, with the expected renal outcome >18 years. However, if the J-score is ≧16, the predicted renal outcome is poor, with the expected renal outcome <2 years. We believe the findings in this study may contribute, in some measure, to a better outcome for patients, affecting diagnosis, choice of treatment strategy, and patients’ life plans.

## Supporting information

S1 Fig Ability to predict 10-year renal outcome with and without pathological score. (DOCX)

S1 Table Definition of JRPS pathological findings. (DOCX)

S2 Table Distribution of pathological findings by cohort set. (DOCX)

S3 Table Correlation coefficient among clinical and pathological factors. (DOCX)

S4 Table Distribution of the new pathological score (J-score) by levels of albuminuria and renal function. (DOCX)

S5 Table Comparison of predictability of 10-year renal outcome in patients with mild albuminuria or with preserved renal function. (DOCX)
