# Supplementary material for: A new pathological scoring system by the Japanese classification to predict renal outcome in diabetic nephropathy
Source: PLoS One. 2018 Feb 6;13(2):e0190923. doi: 10.1371/journal.pone.0190923 (PMC5800536; doi:10.1371/journal.pone.0190923)
Supplement: S1 Fig — (DOCX) [file pone.0190923.s001.docx]

Supplementary Figure 1: Ability to predict 10-year renal outcome with and without pathological score

**Model 1**

0.00

0.25

0.50

0.75

1.00

Sensitivity

0.00

0.25

0.50

0.75

1.00

1 - Specificity


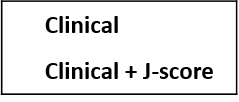


0.00

0.25

0.50

0.75

1.00

Sensitivity

0.00

0.25

0.50

0.75

1.00

1 - Specificity

**Model 2**
