# Supplementary material for: A new pathological scoring system by the Japanese classification to predict renal outcome in diabetic nephropathy
Source: PLoS One. 2018 Feb 6;13(2):e0190923. doi: 10.1371/journal.pone.0190923 (PMC5800536; doi:10.1371/journal.pone.0190923)
Supplement: S1 Table — (DOCX) [file pone.0190923.s002.docx]

Supplementary table 1: Definition of JRPS pathological findings

| Pathological findings | | Score | Definition of score |
| --- | --- | --- | --- |
| Glomerular lesions | Diffuse lesion  (mesangial expansion) | 0-3 | 0 normal or mild mesangial expansion  1 mesangial expansion≦capillary lumen  2 mesangial expansion=capillary lumen  3 mesangial expansion≧capillary lumen |
|  | Nodular lesion | 0,1 | 0 (note detected), 1 (detected) |
|  | Subendothelial space widening (double contour of basement membrane) | 0-3 | Double contour basement membrane (%) (determined in peripheral capillary of the most severe glomerulus)  0 (<10%), 1 (10-25%), 2 (25-50%), 3(≧50%) |
|  | Exudative lesion | 0,1 | 0 (not detected), 1 (detected) |
|  | Polar vasculosis | 0,1 | 0 (not detected), 1 (detected) |
|  | Global glomerulosclerosis | % | Number of glomerulosclerosis/ all glomerulus |
|  | Segmental glomerulosclerosis | % | Number of glomerulosclerosis/ all glomerulus |
|  | Glomerulomegaly | 0,1 | Glomeruli >250μm in diameter  0 (not detected), 1 (detected) |
| Interstitial lesions | Interstitial fibrosis and tubular atrophy (IFTA) | 0-3 | 0 (no IFTA), 1(<25%), 2(25-50%), 3(≧50%) |
|  | Interstitial inflammation | 0-3 | 0(no cell infiltration), 1(<25%), 2(25-50%), 3(≧50%) |
| Vascular lesions | Arteriolar hyalinosis | 0-3 | 0 (no hyalinosis), 1(one or more partial arteriolar hyalinosis), 2(approximately 50% hyalinosis), 3(more than 50% hyalinosis, or penetrating hyalinosis) |
|  | Intimal thickening | 0-2 | 0 (no intimal thickening), 1(intimal thickness/media thickness <1), 2(intimal thickening and intimal thickness/media thickness≧1) |
