# Supplementary material for: A new pathological scoring system by the Japanese classification to predict renal outcome in diabetic nephropathy
Source: PLoS One. 2018 Feb 6;13(2):e0190923. doi: 10.1371/journal.pone.0190923 (PMC5800536; doi:10.1371/journal.pone.0190923)
Supplement: S2 Table — (DOCX) [file pone.0190923.s003.docx]

Supplementary table 2: Distribution of pathological findings by cohort set

|  |  |  | **Total** | |  | **Training set** | | **Test set** | | **p** |
| --- | --- | --- | --- | --- | --- | --- | --- | --- | --- | --- |
|  | **n** |  | **493** | |  | **326** | | **167** | |  |
|  |  |  | JRPS | RPS |  | JRPS | RPS | JRPS | RPS |  |
| Glomerular lesions | |  |  |  |  |  |  |  |  |  |
|  | *RPS* | *I* |  | *25%* |  |  | *24%* |  | *29%* | 0.07 |
|  |  | *IIA* |  | *11%* |  |  | *14%* |  | *5%* |  |
|  |  | *IIB* |  | *15%* |  |  | *19%* |  | *7%* |  |
|  |  | *III* |  | *35%* |  |  | *30%* |  | *43%* |  |
|  |  | *IV* |  | *14%* |  |  | *13%* |  | *16%* |  |
|  | Diffuse lesion | 0 | 6% |  |  | 6% |  | 8% |  | 0.07 |
|  |  | 1 | 26% |  |  | 22% |  | 33% |  |  |
|  |  | 2 | 22% |  |  | 23% |  | 20% |  |  |
|  |  | 3 | 46% |  |  | 49% |  | 40% |  |  |
|  | Nodular lesion |  | 36% |  |  | 37% |  | 34% |  | 0.45 |
|  | GBM doubling | 0 | 49% |  |  | 51% |  | 46% |  | <0.001 |
|  |  | 1 | 39% |  |  | 31% |  | 54% |  |  |
|  |  | 2 | 8% |  |  | 13% |  | 0% |  |  |
|  |  | 3 | 3% |  |  | 5% |  | 0% |  |  |
|  | Mesangiolysis |  | 39% |  |  | 45% |  | 29% |  | 0.001 |
|  | Polar vasculosis | | 69% |  |  | 64% |  | 78% |  | 0.002 |
|  | Glomerulomegary | | 30% |  |  | 34% |  | 23% |  | 0.01 |
|  | Exudative lesion | | 62% | |  | 53% | | 82% | | <0.001 |
| IFTA | |  |  |  |  |  |  |  |  |  |
|  |  | 0 | 10% | |  | 10% | | 11% | | 0.82 |
|  |  | 1 | 31% | |  | 30% | | 33% | |  |
|  |  | 2 | 30% | |  | 31% | | 28% | |  |
|  |  | 3 | 29% | |  | 29% | | 28% | |  |
| Interstitial inflammation | | |  |  |  |  |  |  |  |  |
|  | *RPS* | *0* |  | *13%* |  |  | *10%* |  | *19%* | <0.001 |
|  |  | *1* |  | *66%* |  |  | *73%* |  | *53%* |  |
|  |  | *2* |  | *21%* |  |  | *17%* |  | *28%* |  |
|  | JRPS | 0 | 16% |  |  | 15% |  | 19% |  | 0.02 |
|  |  | 1 | 54% |  |  | 58% |  | 47% |  |  |
|  |  | 2 | 18% |  |  | 18% |  | 17% |  |  |
|  |  | 3 | 12% |  |  | 9% |  | 18% |  |  |
| Arteriolar hyalinosis | | |  |  |  |  |  |  |  |  |
|  | *RPS* | *0* |  | *8%* |  |  | *9%* |  | *5%* | *0.04* |
|  |  | *1* |  | *27%* |  |  | *24%* |  | *34%* |  |
|  |  | *2* |  | *65%* |  |  | *67%* |  | *61%* |  |
|  | JRPS | 0 | 8% |  |  | 9% |  | 5% |  | 0.14 |
|  |  | 1 | 25% |  |  | 22% |  | 30% |  |  |
|  |  | 2 | 20% |  |  | 21% |  | 20% |  |  |
|  |  | 3 | 47% |  |  | 48% |  | 45% |  |  |
| Arteriosclerosis | |  |  |  |  |  |  |  |  |  |
|  |  | 0 | 15% | |  | 19% | | 5% | | <0.001 |
|  |  | 1 | 47% | |  | 46% | | 49% | |  |
|  |  | 2 | 38% | |  | 34% | | 45% | |  |

RPS, classification by renal pathology society (reference 9)

JRPS, classification by Japan renal pathology society (reference 13)

GBM, glomerular basement membrane; IFTA, interstitial fibrosis and tubular atrophy
