# Supplementary material for: A new pathological scoring system by the Japanese classification to predict renal outcome in diabetic nephropathy
Source: PLoS One. 2018 Feb 6;13(2):e0190923. doi: 10.1371/journal.pone.0190923 (PMC5800536; doi:10.1371/journal.pone.0190923)
Supplement: S3 Table — (DOCX) [file pone.0190923.s004.docx]

Supplementary table 3: Correlation coefficient among clinical and pathological factors

Abbreviations: eGFR, estimated glomerular filtration rate; BP, blood pressure; BMI, body mass index; HbA1c, hemoglobin A1c; DMR, diabetic retinopathy; U-Alb, urinary albumin; U-RBC, urinary red blood cells; Diffuse, diffuse lesion; Exudative, exudative lesions; MLysis, mesangiolysis; Polar, polar vasculosis; Mega, glomerulomegarly; IFTA, interstitial fibrosis and tubular atrophy; Int, Interstitial inflammation; Hya, Arteriolar hyalinosis.

Correlation higher than 0.3 was shown in a bold letter
