# Supplementary material for: A new pathological scoring system by the Japanese classification to predict renal outcome in diabetic nephropathy
Source: PLoS One. 2018 Feb 6;13(2):e0190923. doi: 10.1371/journal.pone.0190923 (PMC5800536; doi:10.1371/journal.pone.0190923)
Supplement: S4 Table — (DOCX) [file pone.0190923.s005.docx]

Supplementary table 4: Distribution of the new pathological score (J-score) by levels of albuminuria and renal function

| Albuminuria (mg/gCre) | <30 | 30-299 | ≧300 | p-value |
| --- | --- | --- | --- | --- |
| J-score | 8.5 [2-10] | 11 [8-12] | 13 [11-16] | <0.001 |
| CKD stage | **G1, 2** | **G3** | **G4, 5** |  |
| J-score | 10 [7-13] | 13 [11-16] | 14 [12-17] | <0.001 |

*Median and interquartile range

CKD, chronic kidney disease
