# Supplementary material for: A new pathological scoring system by the Japanese classification to predict renal outcome in diabetic nephropathy
Source: PLoS One. 2018 Feb 6;13(2):e0190923. doi: 10.1371/journal.pone.0190923 (PMC5800536; doi:10.1371/journal.pone.0190923)
Supplement: S5 Table — (DOCX) [file pone.0190923.s006.docx]

Supplementary table 5: Comparison of predictability of 10-year renal outcome in patients with mild albuminuria or with preserved renal function.

| **Albuminuria <300 mg/gCre** | **C-statistics (95% CI)** | **NRI (SE,** *p-value***)** | **IDI (SE,** *p-value***)** |
| --- | --- | --- | --- |
| **Model 1 (eGFR, Alb)** |  |  |  |
| Clinical | 0.6158 (0.4818, 0.7498) |  |  |
| Clinical + J-score | 0.6788 (0.5555, 0.8022) | 0.249 (0.253, *0.33*) | 0.021 (0.017, *0.22*) |
| Clinical + D-score | 0.7346 (0.5067, 0.9625) | 0.839 (0.371, *0.03*) | 0.085 (0.051, *0.10*) |
|  |  |  |  |
| **Model 2 (7 factors)** |  |  |  |
| Clinical | 0.6885 (0.5482, 0.8288) |  |  |
| Clinical + J-score | 0.7432 (0.6126, 0.8737) | 0.379 (0.265*, 0.15*) | 0.022 (0.016, *0.16*) |
| Clinical + D-score | 0.7635 (0.6098, 0.9172) | 0.727 (0.375*, 0.05*) | 0.135 (0.071, *0.06*) |

| **eGFR≧60 mL/min/1.73m^2^** | **C-statistics (95% CI)** | **NRI (SE, p-value)** | **IDI (SE, p-value)** |
| --- | --- | --- | --- |
| **Model 1 (eGFR, Alb)** |  |  |  |
| Clinical | 0.6815 (0.6006, 0.7624) |  |  |
| Clinical + J-score | 0.7565 (0.6793, 0.8338) | 0.519 (0.171, 0.003) | 0.057 (0.018, 0.002) |
| Clinical + D-score | 0.8292 (0.7178, 0.9407) | 0.840 (0.261, 0.001) | 0.069 (0.033, 0.03) |
|  |  |  |  |
| **Model 2 (7 factors)** |  |  |  |
| Clinical | 0.6927 (0.6059, 0.7795) |  |  |
| Clinical + J-score | 0.7602 (0.6778, 0.8425) | 0.524 (0.181, 0.002) | 0.077 (0.022, <0.001) |
| Clinical + D-score | 0.8385 (0.7067, 0.9702) | 0.648 (0.272, 0.02) | 0.100 (0.039, 0.01) |

Clinical model 1: estimated glomerular filtration rate (eGFR) and albuminuria category

Clinical model 2: age, sex, eGFR, blood pressure, body mass index, hemoglobin A1c, and albuminuria category

NRI, net reclassification improvement; IDI, integrated discrimination improvement; SE, standard error.
